# Case Report: Challenges in the diagnosis and treatment of cardiac amyloidosis: a case misdiagnosed as dual cardiac amyloidosis

**DOI:** 10.3389/fcvm.2025.1692380

**Published:** 2025-11-24

**Authors:** Menghuai Ma, Limin Liu, Xi Liu, Xiang Cai, Wenhui Yang

**Affiliations:** 1 Department of Cardiology, Fuwai Yunnan Hospital, Chinese Academy of Medical Sciences, Affiliated Cardiovascular Hospital of Kunming Medical University, Kunming, China; 2 Department of Cardiology, Fuwai Hospital, National Center for Cardiovascular Diseases, Chinese Academy of Medical Sciences and Peking Union Medical College, Beijing, China

**Keywords:** cardiac amyloidosis, transthyretin amyloidosis, immunoglobulin light chain amyloidosis, ^99m^Technetium pyrophosphate, diagnosis

## Abstract

Cardiac amyloidosis (CA) is a pathologic condition characterized by the cardiac deposition of insoluble misfolded proteins, often resulting in nonspecific symptoms that complicate diagnosis. Although advances in diagnostic techniques have improved the diagnostic rates, accurate identification of amyloid fibril deposits remains challenging. Here, we present a 52-year-old Chinese woman who was initially diagnosed with both wild-type cardiac transthyretin amyloidosis (ATTR-CA) and lambda cardiac light-chain amyloidosis (AL-CA) after the standardized diagnostic workup. Subsequent liquid chromatography-tandem mass spectrometry, however, identified lambda light chains as the primary amyloidogenic protein, confirming a final diagnosis of lambda AL-CA. The diagnostic process was further complicated when a review of ^99m^Tc-pyrophosphate scintigraphy turned strongly positive after an eleven-month interval. Following the patient's refusal of chemotherapy, her heart failure progressively worsened, requiring the support of vasoactive and inotropic drugs, and she ultimately succumbed to cardiogenic shock 17 months after initial presentation. This case underscores the diagnostic challenges and necessity of sophisticated techniques for accurately typing CA.

## Introduction

Cardiac amyloidosis (CA) is a progressive disease caused by amyloid fibril deposition in the cardiomyocyte interstitium, leading to heart failure with a restrictive physiology pattern. Although over 30 amyloidogenic proteins have been identified, amyloid transthyretin (ATTR) and immunoglobulin light chains (AL) account for >98% of diagnosed CA cases (1). Accurate subtyping is crucial, as their optimal treatments differ significantly between these two forms. Despite advances in diagnostic techniques, the subtyping of CA remains challenging. Previous reports have indicated that ATTR amyloidosis could coexist with monoclonal gammopathy of undetermined significance (MGUS) (2), and cardiac light-chain amyloidosis (AL-CA) patients may show positive on ^99m^Tc-pyrophosphate (^99m^Tc-PYP) scintigraphy (1). Additionally, co-deposition of AL and ATTR has been reported (3–5), and a report describe AL-CA patients who later developed ATTR cardiac amyloidosis (ATTR-CA) (6). Here, we present a diagnostically complex CA case initially considered as dual amyloidosis (ATTR-CA and AL-CA), in which lambda AL-CA was ultimately confirmed by liquid chromatography-tandem mass spectrometry (LC-MS/MS). This case underscores the diagnostic and therapeutic challenges of this disorder.

## Case report

In May 2023, a 52-year-old woman was admitted to our hospital with acute decompensated heart failure secondary to volume overload (New York Heart Association functional, NYHA class Ⅲ). She had a one-year history of exertional dyspnea, poor appetite, abdominal distension and upper limb numbness. Physical examination revealed bilateral pulmonary rales, jugular venous congestion, and massive skin ecchymosis. A transthoracic echocardiogram (TTE) performed one year prior to admission showed a left ventricular ejection fraction (LVEF) of 54% and concentric left ventricular (LV) hypertrophy (mean wall thickness 12 mm). Laboratory testing revealed persistently elevated high-sensitivity troponin I (hs-TNI) levels (0.07–0.09 ng/mL). Coronary angiography showed only 50% stenosis of the first diagonal branch.

**Figure 1:**
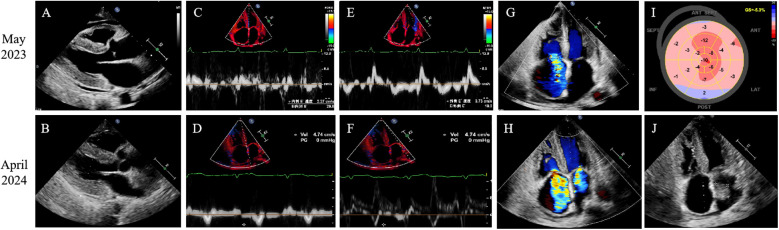
Transthoracic echocardiogram images. **(A,B)** Parasternal long-axis views revealed LV thickness of 13 mm and right ventricular thickness of 7 mm. **(C–F)** Transmitral Doppler imaging revealed decreased Sep e′ and Lat e′. **(G,H)** The colour Doppler flow imaging showed tricuspid regurgitation. **(I)** Left ventricular global longitudinal strain analysis indicated strain of 5.3%, with prominent apical sparing. **(J)** The apical four-chamber view showed a left atrial thrombus (29*25 mm).

**Figure 2:**
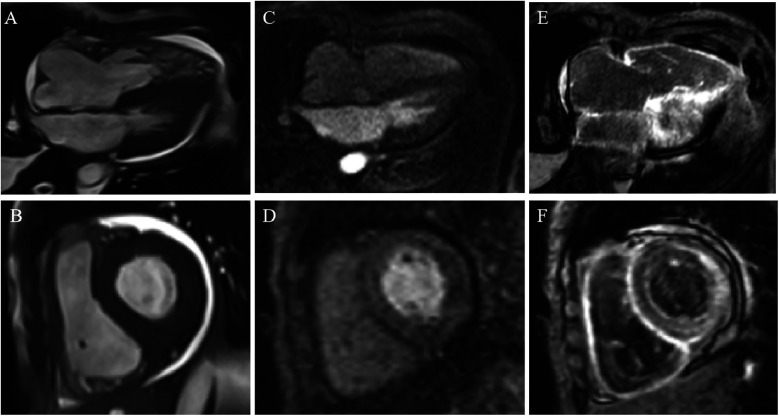
Cardiac magnetic resonance images. **(A,B)** The images of cine scan. **(C,D)** The images of first-pass myocardial perfusion scan. **(E,F)** Late gadolinium enhancement (LGE) imaging showed diffuse LGE.

**Figure 3:**
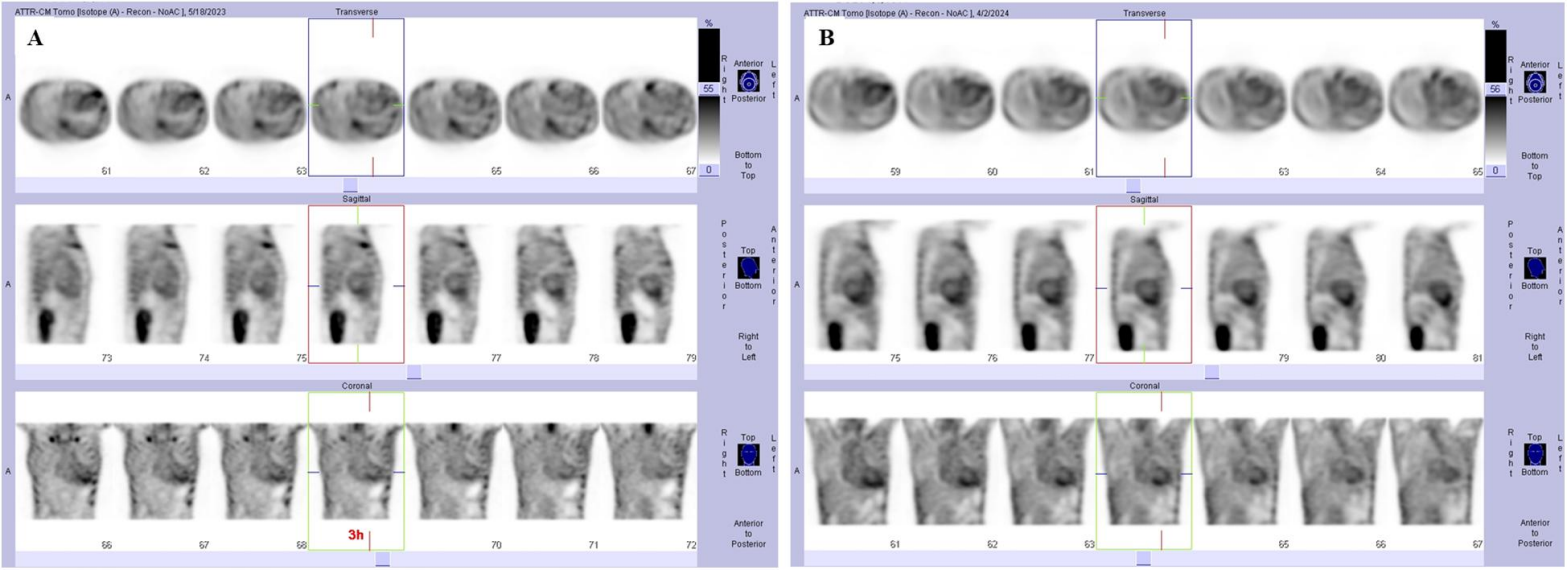
Nuclear ^99m^Tc-pyrophosphate scintigraphy images. **(A)** The imaging was graded visually as grade 2 uptake, with a heart/contralateral lung (H/CL) ratio of 1.27. **(B)** The imaging was interpreted as grade 3 uptake, with a H/CL ratio of 1.35.

On admission, electrocardiography (ECG) showed low QRS voltage, and pseudonecrosis Q waves. Laboratory testing revealed elevated levels of hs-TNI at 0.107 ng/mL and N-terminal pro-hormone B-type natriuretic peptide (NT-proBNP) at 5,669 pg/mL. Chest x-ray showed pulmonary oedema and pleural effusion. The TTE showed that LVEF decreased to 35%, myocardial granular sparkling, diffuse thickened biventricular walls (Figure 1A), severe diastolic dysfunction with a restrictive pattern (Figures 1C,E), mild tricuspid regurgitation (Figure 1G) and relative apical-sparing of LV global longitudinal strain (Figure 1I). Cardiac magnetic resonance (CMR) imaging strongly suggested the presence of CA, characterized by biventricular hypertrophy (Figures 2A,B), diastolic and systolic dysfunction (LVEF 41%), reduced first-pass myocardial perfusion (Figures 2C,D), with patchy and global subendocardial late gadolinium enhancement (LGE) involving biventricular and atrial walls (Figures 2E,F). Serum free light chain found a diminished serum kappa/lambda ratio of 0.17. Serum/urine immunofixation electrophoresis (IFE) detected lambda monoclonal protein. Bone marrow biopsy exhibited 4% plasma cells without evidence of plasma cell dyscrasia or lymphoproliferative disorder. ^99m^Tc-PYP showed enhanced cardiac uptake (Perugini grade 2) (Figure 3A), while the genetic analysis identified no pathogenic variants. Therefore, AL-CA, MGUS and wild-type ATTR-CA were all suspected.

**Figure 4:**
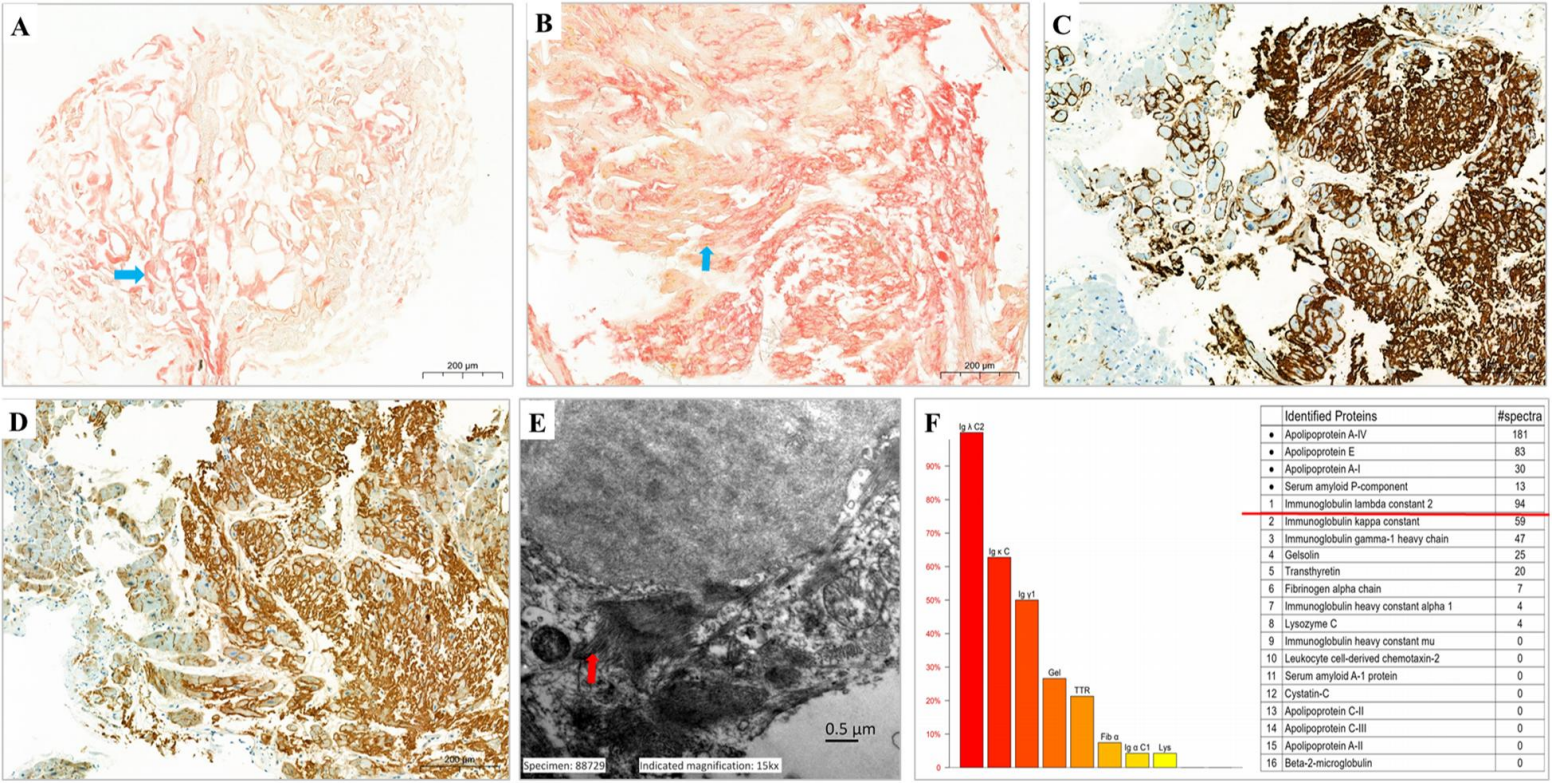
Imaging of abdominal and endomyocardial biopsy. **(A,B)** Congo-red staining abdominal adipose **(A)** and endomyocardial **(B)** biopsy specimen was strongly positive (blue arrow). **(C,D)** Immunohistology images of anti-lambda staining **(C)** and anti-transthyretin staining **(D,E)** Electron microscope revealed the amyloid fibrils were deposited in the myocardial interstitium (red arrow). **(F)** Result of proteomic analysis by liquid chromatography-tandem mass spectrometry showed a predominance of spectral counts for light-chain lambda peptides.

To establish a definitive diagnosis, the abdominal adipose biopsy and endomyocardial biopsy (EMB) were performed. Congo red staining demonstrated amyloid fibril deposition around abdominal adipocytes and within the myocardial interstitium (Figures 4A,B), exhibiting characteristic apple-green birefringence under polarized light. Electron microscope revealed abundant unbranched and rigid amyloid fibrils within the myocardial interstitium (Figure 4E), without evidences of large mitochondria, glycogen accumulation, or myelin-like structures. Immunohistochemical staining revealed positive lambda light chains and transthyretin in distinct areas of the specimen, while kappa light chains and amyloid A were negative (Figures 4C,D). The pathology established a dual diagnosis of ATTR-CA and lambda AL-CA.

In addition to diuretic therapy and oral tafamidis, she was initiated on chemotherapy (including daretumab, bortezomib, cyclophosphamide and dexamethasone), which was discontinued following acute heart failure during the first course. Despite treated with diuresis and tafamidis, her heart failure progressed, requiring hospital readmission. LC-MS/MS was performed to confirm the diagnosis, and identified lambda AL as the main amyloidogenic proteins in the sample (Figure 4F). Consequently, the diagnosis of AL-CA was established, and tafamidis was removed from medication management.

**Figure 5:**
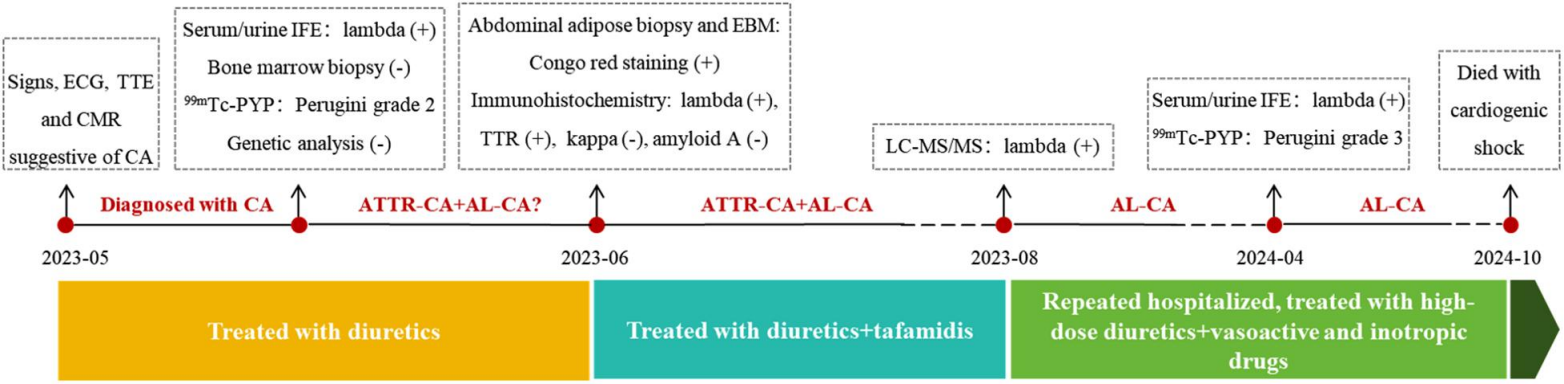
The timeline of diagnosis and treatment.

Progressive amyloid deposition led to end-stage heart failure with multiorgan dysfunction (significant weight loss, hepatic and renal dysfunction), complicated by severe mitral/tricuspid regurgitation and atrial thrombosis (Figure 1H,J), necessitating repeated hospitalizations. Laboratory tests found that hs-TNI increased to 0.834 ng/mL, and NT-proBNP rose to 32,213 pg/mL in April 2024. A review of the ^99m^Tc-PYP scan was conducted and showed grade 3 myocardial uptake (Figure 3B). Serum/urine IFE reconfirmed the presence of lambda monoclonal protein. The patient declined additional EMB or LC-MS/MS. As the CA progressed, the patient developed refractory heart failure and cardiogenic shock. The combination of dopamine and natrecor, along with high-dose diuretics, failed to reduce the volume overload. After adding levosimendan or milrinone, the patient's daily urinary output increased from 1,500 mL to over 2,000 mL and the symptoms were rapidly relieved. Finally, the patient was discharged in NYHA class Ⅱ, and received treatment with high-dose diuretics, including torasemide 120 mg, furosemide 80 mg, bumetanide 6 mg, hydrochlorothiazide 50 mg and tolvaptan 7.5 mg daily. In September 2024, the LVEF declined to 25% and paroxysmal ventricular tachycardia was detected on Holter ECG. She ultimately succumbed to cardiogenic shock in October 2024. A timeline of diagnostic results and treatment adjustments is presented in Figure 5.

## Discussion

In this report, the patient was initially considered concomitant wild-type ATTR-CA and lambda AL-CA, but the eventual diagnosis of lambda AL-CA was confirmed by LC-MS/MS, yet the results of the diagnostic workup were contradictory. As the patient declined chemotherapy, her heart failure progressed persistently, and she ultimately passed away 17 months post-diagnosis. This case has important educational implications for managing CA, particularly regarding the significance of accurate subtyping. Diagnostic delays and misdiagnosis are common and contribute to increased symptom burden, cumulative organ damage, worse prognosis, and higher early mortality (7). In the present case, diagnostic delay led to progressive organ impairment, which limited tolerance to standard-of-care chemotherapy.

Beyond early diagnosis, this report highlights the critical need for precise amyloid fibril typing given divergent treatment pathways. Non-invasive methods reveal some differences between AL-CA and ATTR-CA: ATTR-CA often exhibits less frequent low voltage on ECG, more common transmural LGE, higher LV mass, and greater intracellular volume on CMR (8). However, conclusive classification of amyloid fibril requires a thorough assessment. Current guidelines suggest performing initial monoclonal proteins test, and patients with abnormal findings should receive bone marrow biopsy and EMB (1). When a monoclonal protein is identified by serum free light chain assay and serum/urine IFE, the negative predictive value for excluding AL amyloidosis reaches nearly 99% (9). In the absence of monoclonal proteins, a Perugini Score ≥2 on ^99m^Tc-PYP is highly specific (100%) for ATTR-CA diagnosis, and may obviate the need for EMB (1). In some clinical scenarios, concurrent ^99m^Tc-PYP and monoclonal proteins testing may be warranted, particularly when the ATTR-CA cannot be excluded. However, prior studies found that >20% of AL-CA patients showed Perugini Score ≥2 on ^99m^Tc-PYP (9), and up to 20%–40% patients with ATTR-CA had coexisting MGUS (2). The concurrent ATTR and AL amyloidosis in individual patients have also been reported (3–5). Therefore, EMB is often necessary as the diagnostic gold standard (1), particularly in cases where both monoclonal proteins testing and ^99m^Tc-PYP yield positive results. In our patient, the positive results from both IFE (lambda) and 99mTc-PYP (Perugini grade 2) prompted the performance of an EMB. The immunohistochemical analysis revealed strong positive staining for both ATTR and AL. She was initially diagnosed as double CA(ATTR and AL), however, the immunohistochemistry may also yield false positive results (10).

To overcome the limitations of existing methods, semi-quantitative LC-MS/MS has been introduced as an advanced diagnostic tool. This technique involves the microdissection of proteins from histological sections, followed by tryptic digestion and LC-MS/MS analysis. It quantifies the relative abundance of amyloid fibrils, enabling highly accurate amyloid subtyping with near 100% sensitivity and specificity (10). Studies have demonstrated its superiority over immunohistochemistry in confirming amyloid fibril type (10, 11). It was LC-MS/MS that ultimately confirmed the AL-CA diagnosis in our patient, whose rapidly progressive course leading to heart failure-related death within two years further supports this diagnosis. Notably, unlike prior reports that used LC-MS/MS to confirm dual amyloidosis by targeting immunohistochemistry-positive sites (3, 4), our sample was analyzed without immunohistochemical guidance—a methodological difference that may have impacted the findings.

Most notably, the patient's follow-up ^99m^Tc-PYP scan showed progression to Perugini grade 3. The mechanism behind this finding, although not fully understood, may be clarified by existing theories on ^99m^Tc-PYP uptake. One prevailing theory posits a calcium-dependent binding mechanism (12). Given its high affinity for calcium—a ion often enriched in damaged tissues—^99m^Tc-PYP can yield positive results in any condition featuring dystrophic calcification, such as valvular calcification, myocardial infarction, rib fractures, or hydroxychloroquine cardiotoxicity (9). Supporting this, a recent case of uremic cardiomyopathy showed a strongly positive ^99m^Tc-PYP scan and revealed numerous myocardial calcifications, primarily located in areas of interstitial fibrosis on EMB (13). Compared to AL-CA, ATTR-CA typically exhibits a greater density of microcalcifications, which may account for the stronger ^99m^Tc-PYP avidity in ATTR-CA (12). However, some AL-CA patients can demonstrate densities of microcalcifications comparable to the ATTR-CA, leading to false-positive ^99m^Tc-PYP scans (9). Another hypothesis relates ^99m^Tc-PYP binding to the chronicity of cardiac amyloid deposition. The typically more indolent course of ATTR-CA allows for longer deposition periods compared to AL-CA. Additionally, structural analyses suggested that exposed acidic pairs on amyloid fibril surfaces may promote calcification and tracer uptake, with this property varying by fibril type (14). ATTR fibrils, with their highly stable amino acid sequences, generally bind 99mTc-PYP, while in AL the opposite is true. Therefore, we speculate that the AL amyloid fibrils in our patient may possess exposed acidic pairs capable of binding 99mTc-PYP. The progressive amyloid deposition, fibrosis, and calcification in her advancing AL-CA could thereby intensify radiotracer uptake. Although rare, the subsequent emergence of ATTR-CA was also considered, as reported in a patient who developed ATTR-CA years after successful treatment for AL-CA (6).

The exclusion of chemotherapy due to patient's refusal led to a rapid clinical deterioration, culminating in the need for vasoactive and inotropic support. While this patient primarily manifests as a restrictive cardiomyopathy characterized by diastolic dysfunction and elevated filling pressures, her symptoms were mitigated by milrinone and levosimendan. Levosimendan is known to enhance myocardial contractility and vasodilation, thereby increasing cardiac output, promoting diuresis, and relieving congestion. Accordingly, milrinone improves LV diastolic function, reduces pulmonary artery pressure, and promotes systemic vasodilation, positioning it as a potential therapeutic strategy for CA patients with acute decompensation and hypoperfusion. A small-sample study has suggested the safety of these inotropes in such inotrope-dependent CA patients (15). However, robust evidence supporting inotropes utilization in CA remains lacking.

This case highlights the diagnostic challenges associated with persistent myocardial uptake of ^99m^Tc-PYP and the potential for false-positive results in immunohistochemistry for AL-CA. It emphasizes the need for LC-MS/MS to achieve definitive subtyping. Additionally, it suggests that inotropes may be safe and effective for patients with CA who are experiencing end-stage heart failure.

## Data Availability

The original contributions presented in the study are included in the article. Further inquiries can be directed to the corresponding author.
